# Principles and Biomedical Applications of Self-Assembled Peptides: Potential Treatment of Type 2 Diabetes Mellitus

**DOI:** 10.3390/pharmaceutics16111442

**Published:** 2024-11-12

**Authors:** Alireza Mohammad Karim

**Affiliations:** Nanoscience Centre, Department of Engineering, University of Cambridge, 11 J. J. Thomson Avenue, Cambridge CB3 0FF, UK; am2633@cantab.ac.uk

**Keywords:** molecular self-assembly, nanomedicine, nanofibril, nanotherapeutics, type 2 diabetes mellitus

## Abstract

Type 2 diabetes mellitus (T2DM) is the most prevalent metabolic disorder worldwide. There have been tremendous efforts to find a safe and prolonged effective therapy for its treatment. Peptide hormones, from certain organisms in the human body, as the pharmaceutical agents, have shown outstanding profiles of efficacy and safety in plasma glucose regulation. Their therapeutic promises have undergone intensive investigations via examining their physicochemical and pharmacokinetic properties. Their major drawback is their short half-life in vivo. To address this challenge, researchers have recently started to apply the state-of-the-art molecular self-assembly on peptide hormones to form nanofibrillar structures, as a smart nanotherapeutic drug delivery technique, to tremendously enhance their prolonged bioactivity in vivo. This revolutionary therapeutic approach would significantly improve patient compliance. First, this review provides a comprehensive summary on the pathophysiology of T2DM, various efforts to treat this chronic disorder, and the limitations and drawbacks of these treatment approaches. Next, this review lays out detailed insights on various aspects of peptide self-assembly: adverse effects, potential applications in nanobiotechnology, thermodynamics and kinetics of the process, as well as the molecular structures of the self-assembled configurations. Furthermore, this review elucidates the recent efforts on applying reversible human-derived peptide self-assembly to generate highly organized smart nanostructured drug formulations known as nanofibrils to regulate and prolong the bioactivity of the human gut hormone peptides in vivo to treat T2DM. Finally, this review highlights the future research directions to advance the knowledge on the state-of-the-art peptide self-assembly process to apply the revolutionary smart nanotherapeutics for treatment of chronic disorders such as T2DM with highly improved patient compliance.

## 1. Self-Assembly of Peptides and Its Biomedical Applications

Peptides exhibit an outstanding role as a therapeutic agent due to their high precision and safety profile. Peptides show promising application in the treatment of numerous diseases such as cardiovascular and metabolic complications [1,2,3,4,5,6,7,8,9,10,11,12,13,14,15,16,17,18,19,20,21,22,23,24,25,26,27,28,29,30,31,32,33,34,35,36,37,38,39,40,41,42,43,44,45,46,47,48,49,50,51,52,53,54,55,56,57,58,59,60,61,62,63,64,65,66,67,68,69,70,71,72,73,74,75,76,77,78,79,80,81,82,83,84,85,86,87,88,89,90,91,92,93,94,95,96,97,98,99,100,101]. Despite their outstanding potential in therapy, their use is challenging due to their extremely poor in vivo bioactivity through high chemical instability, extreme vulnerability to proteolytic degradation via enzymes, high clearance profile, and very short plasma half-life [99,100,102].

To address these challenges, self-assembly of peptides into highly organized and stable nanostructures known as nanofibrils is applied [1,2,3,4,5,6,7,8,9,10,11,12,13,14,15,16,17,18,19,20,21,22,23,24,25,26,27,28,29,30,31,32,33,34,35,36,37,38,39,40,41,42,43,44,45,46,47,48,49,50,51,52,53,54,55,56,57,58,59,60,61,62,63,64,65,66,67,68,69,70,71,72,73,74,75,76,77,78,79,80,81,82,83,84,85,86,87,88,89,90,91,92,93,94,95,96,97,98,101,103,104]. This fascinating non-specific physio-chemical process is accomplished via the intrinsic tendency of the peptide amino acid sequences to self-assemble into highly β-sheet-rich nanofibrils through the formation of a hydrogen bond network [105,106,107,108].

It has been shown that upon subcutaneous administration of the self-assembled GLP-1/glucagon peptide nanofibrils in rodents, the GLP-1/glucagon native peptides were released from the nanofibrils into the blood stream [109]. Self-assembled peptides were significantly considered for numerous advanced functional biomedical applications including bacterial coatings and scaffolds [110,111]. Self-assembled peptides have been tremendously used for many clinical and pharmaceutical purposes including advanced drug delivery systems such as peptide micro-capsules, peptide nano-capsules, and tissue engineering as hydrogels [112,113,114,115,116]. Self-assembled human peptides have shown outstanding biocompatibility and stability, which make them suitable for prolonged nanotherapeutics for treatment of chronic disorders such as cardiovascular and metabolic complications [112,113,114,115,116]. One of the most prevalent chronic metabolic disorders is type 2 diabetes mellitus (T2DM) [117,118,119,120,121,122,123]. There have been numerous approaches to treat T2DM. However, none of them are sufficiently patient compliant, strongly safe, and effective [124,125,126,127,128,129,130,131,132,133,134,135,136,137,138,139,140].

First, this review addresses the fundamental concepts of the peptide self-assembly process, including its general mechanism, the molecular structure of the formed self-assembled peptides, and thermodynamics and kinetics of the peptide self-assembly process. Next, this review provides a summary of the pathophysiology and potential effective role of self-assembled peptides in biotechnology, including its promising therapeutic role in the treatment of chronic disorders. Afterward, this review addresses the pathophysiology of type 2 diabetes mellitus (T2DM) and current treatment approaches and their drawbacks and limitations for this chronic disorder, which cause low patient compliance. Then, this review highlights the outstanding potential role of gut peptide hormone self-assembly into nanofibrillar structures, which could enhance the patient compliance of treatment of T2DM. Finally, this review addresses the future research directions in this field to achieve the goal, which is to use this innovative treatment approach for patients with chronic disorders, specifically with T2DM.

## 2. What Are Self-Assembled Peptides?

**Figure 1:**
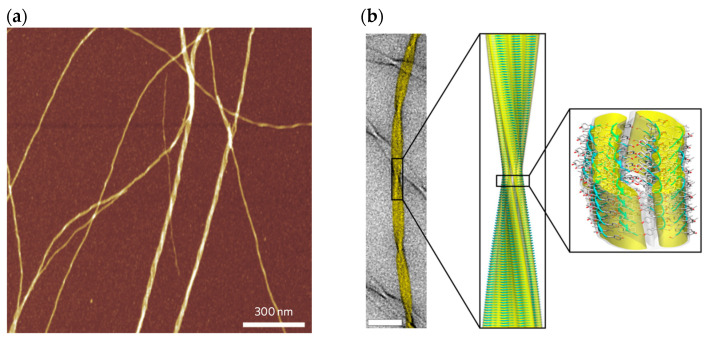

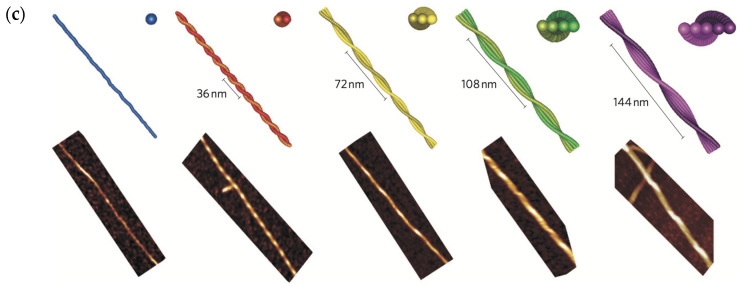
(**a**) Atomic force microscopy (AFM) image of β-lactoglobulin nanofibrils [145]. (**b**) Atomic resolution structure of a nanofibril using the magic angle spinning (MAS) nuclear magnetic resonance (NMR) technique from the cryo-EM image of the nanofibrils: left picture side shows the cryo-EM image of the nanofibrils, center picture shows the MAS NMR atomic resolution of the zoomed-in section of a nanofibril from the cryo-EM image, right picture shows the ribbon-like configuration of the β-sheets in the zoomed-in section of the center picture [144]. (**c**) AFM images of the multistranded twisted ribbon-like nanofibrils with various numbers of filaments [145]. (**b**) Reprinted/adapted with permission from [144]. 2013, Fitzpatrick, A.W., et al. (**a**,**c**) Reprinted/adapted with permission from [145]. 2010, Adamcik, J., et al.

Self-assembled peptides are extremely ordered peptide aggregates in the form of nanofibrillar structures. Self-assembled peptides are known as nanofibrils. Nanofibrils are normally characterized to have a thickness of around 100 to 200 Å in diameter with a general “cross-β-sheet” fundamental structural unit which contain several arrays of β-sheets situated parallel next to each other along the axis of the nanofibril [141,142,143]. Figure 1 shows the atomic force microscopic and cryogenic electron microscopic images of the atomic resolution structure of the cross-β-sheet nanofibrils [144,145].

Nanofibril conformation is regulated by the tendency of the free peptide backbone to create hydrogen bonds between the peptide backbones. Each peptide with an arrowhead pointed β-strand piles on top of the next peptide in a parallel arrangement in which the axis of a peptide backbone is perpendicular to the axis of the nanofibril. Each hydrogen bond is created between an oxygen atom of a carbonyl section of an amino acid and a hydrogen atom of the amine section of another amino acid along the peptide backbone.

**Figure 2:**
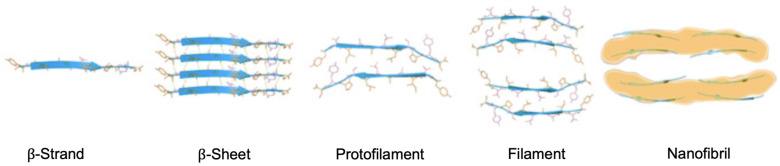
Cross-sectional schematic hierarchy of the peptide self-assembly mechanism towards formation of a nanofibril structure [144]. Each β-strand represents a peptide. Peptides stack together via formation of the backbone–backbone hydrogen bonds to generate a β-sheet. Two cross-β-sheets (anti-parallel β-sheets) line up together via interactions between their amino acid side chains to create a proto-filament. Afterwards, the protofilaments line up next to each other or in an anti-parallel fashion to generate a filament. Finally, the filaments stack up together by interactions between corresponding side chains to form a stable nanofibrillar structure. Reprinted/adapted with permission from [144]. 2013, Fitzpatrick, A.W., et al.

Amino acid side chains rest on either side of the cross-β-sheet. The β-sheet conformation is controlled by the intermolecular forces between similar amino acid side chains, including π–π molecular interactions of aromatic rings, the hydrophobic interactions, and the electrostatic interactions between charged side chains [146]. The core structure of the nanofibril might not be composed of the complete amino acid sequence of the building peptides [141,147]. A schematic illustration of the structure of peptide self-assembly towards a nanofibrillar conformation is shown in Figure 2.

Proto-nanofibrils mainly consist of two to four β-sheets in which the peptides stack together in a parallel fashion within a β-sheet and antiparallel to an opposite β-sheet [141,147,148,149,150,151,152,153,154]. In a cross-β-sheet conformation, the peptides (denoted as arrowhead β-strands) are separated by approximately 4.6 to 4.8 Å. Two opposite β-sheets are separated by approximately 8 to 10 Å [144]. The thickness of the nanofibrils normally is in the order of a few nanometers, and their lengths are in the order of several micrometers [145,152,155,156]. Limitations in the arrangement of side chains and electrostatic forces between them lead to the twist of the peptides as well as the nanofibrils. The degree of twist strongly depends on the amino acid sequence of the peptide, number of β-sheets and their arrangement, and conditions of the solution including pH, temperature, salt type, and salt size [144,149,157,158]. Nanofibril conformation generates a protective and stable space for free peptides in a wide range of physical conditions and can maintain the peptide amino acid sequence from proteolytic degradation of enzymes [159,160]. Due to these factors, nanofibrils are normally highly elastic with a large modulus of elasticity and large bending rigidity [107].

## 3. Peptide Self-Assembly: Thermodynamics and Kinetics

### 3.1. Thermodynamics

**Figure 3:**
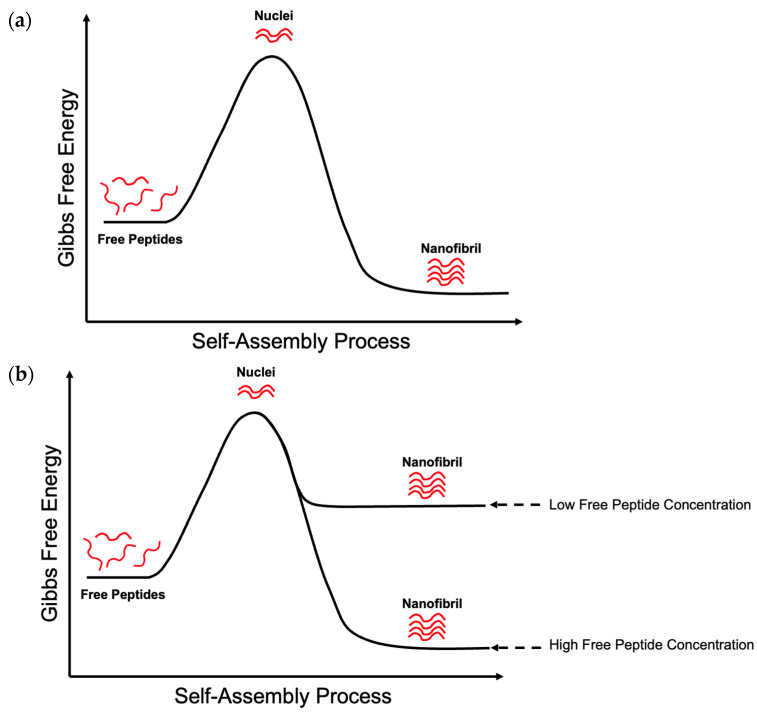
Schematics of the thermodynamics of the peptide self-assembly process to form a nanofibrillar structure. (**a**) Gibbs free energy change via peptide self-assembly into a nanofibril [165]. (**b**) Gibbs free energy change depends on concentration of free peptides in the system [146]. (**a**) Reprinted/adapted with permission from [165]. 2015, Arosio, P., et al. (**b**) Reprinted/adapted with permission from [146]. 2005, Nelson, R., et al.

Peptides can spontaneously self-assemble into various forms of highly organized structures such as twisted nanofibrils, tubular structures, helical nanofibrils, and globular configurations based on the amino acid sequence [159,161,162]. However, it was reported that the formation of an organized self-assembled structure is not related to any specific amino acid sequence. But, it is extremely attributed to the available minimum Gibbs free energy level based on the bio-physio-chemical conditions of the environment [163]. The native state of a peptide is defined by its amino acid sequence and is unique for that specific amino acid sequence. However, the amino acid sequence can be denatured and consequently stretched to generate a cross-β sheet structure with an alternative available minimum Gibbs free energy level which is lower than the native state’s minimum Gibbs free energy level, as sketched in Figure 3a. The nanofibrillar structure, generated by self-assembly of peptides with a specific amino acid sequence, extremely depends on the backbone conformation of the peptide. Peptide concentration, [*M_p_*], has a critical role in the Gibbs free energy curve, which describes the thermodynamics of the fibrillation process, as shown in Figure 3b. At low peptide concentration, free peptides are favored in the self-assembly reaction, in contrast with high peptide concentration, in which nanofibrillar structures are favored to the self-assembly process. The self-assembly process shown in Reaction (1) depicts the generation of nanofibrillar structures with concentration, [*M_f_*], from *l* out of *n* free peptides with concentration, [*M_p_*]:

(1) $$n\;M_{p}\to l\;M_{p}+M_{f}$$

In a peptide self-assembly Reaction (1), at the equilibrium state, a combination of nanofibrillar structures and free peptides coexist. This is attributed to the fact that the self-assembly process involves association and dissociation. In the association process, free peptides associate to create nuclei, and further peptide association leads to the nanofibrillar structures. In the dissociation process, nuclei and preformed nanofibrillar structures dissociate back to free peptides. The difference between Gibbs free energy of free peptides, *G_p_*, and Gibbs free energy of nanofibrillar structures, *G_f_*, is denoted by ∆G, as shown in Equation (2) [146,164]. In Equation (2), *R* is the universal specific gas constant and *T* is the absolute temperature.

(2) $$∆G=G_{f}-G_{p}=R\;T\;\ln\left(\frac{\left[M_{f}\right]}{{\left[M_{p}\right]}^{\left(n-l\right)}}\right)$$

The expression on the right side of Equation (2) denotes the role of free peptide concentration in the Gibbs free energy difference (∆G) at the equilibrium state. At high free peptide concentration, the expression on the right side is negative, showing the reaction favors nanofibrillar structures at the equilibrium. In Equation (2), the quotient describes the ratio of the dissociation rate, *k_off_*, over the association rate, *k_on_*, as noted in Equation (3). The dissociation rate is the rate of conversion from nanofibrillar structure to free peptide conformation. The association rate is the speed of conversion from free peptide conformation to the nanofibrillar structure.

(3) $$∆G=R\;T\;\ln\left(\frac{k_{off}}{k_{on}}\right)$$

Nuclei, consisting of a small number of peptides, should be properly generated to complete the self-assembly process to generate a stable nanofibril. The required Gibbs free energy to form the minimum number of nuclei needed to complete the self-assembly procedure is denoted by Gibbs free energy of formation. The standard Gibbs free energy of nuclei formation, ${∆G}_{associate}$, with the required number of free peptides, is denoted as the sum of standard enthalpy of formation, ${∆H}_{associate}$, and standard entropy of formation, ${∆S}_{associate}$:

(4) $${∆G}_{associate}=G_{n}-G_{p}={∆H}_{associate}-T\;{∆S}_{associate}$$

In Equation (4), the standard Gibbs free energy for formation of nuclei is denoted by the difference between Gibbs free energy of nuclei, $G_{n}$, and Gibbs free energy of peptides, $G_{p}$ [146]. Since Gibbs free energy of nuclei is larger than Gibbs free energy of peptides, the nuclei formation is not spontaneous, and it requires some external stimuli including heat via temperature rise, shear stress by agitation, salt solution, and pH change to initiate nuclei generation.

Once nuclei are created, more peptides are associated to them, leading to further growth of nuclei towards complete nanofibrillar structures [146]. Moreover, the concentration of free peptides incorporated in the process of the formation of nanofibrils determines the direction of the self-assembly process at the equilibrium condition by specifying the relative difference between Gibbs free energy required for generation of nuclei, ${∆G}_{associate}$, and Gibbs free energy for dissociation of nanofibrils to free peptides, ${∆G}_{dissociate}$. At high free peptide concentration, Gibbs free energy needed for nanofibril dissociation towards nuclei and further dissociation to free peptides is much larger than for the process of nanofibril formation, which makes it difficult for nanofibrils to dissociate into free peptides.

### 3.2. Kinetics

**Figure 4:**
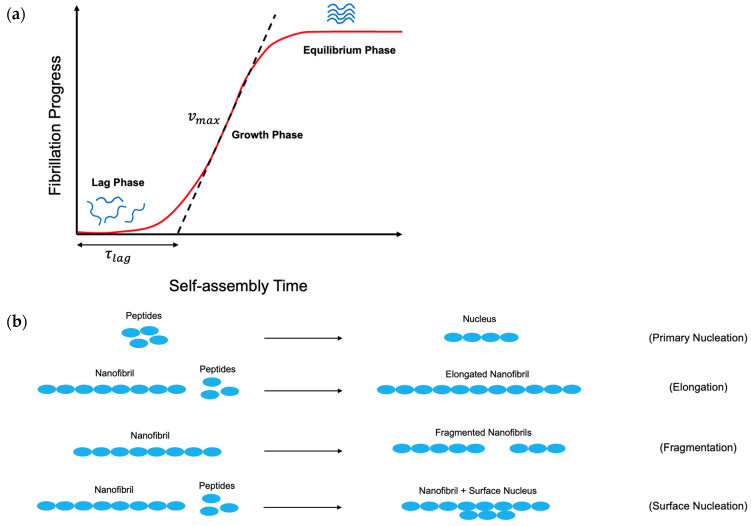
(**a**) Sigmoidal curve to illustrate the kinetics of the peptide self-assembly process generating mature stable nanofibrillar structures [165]. (**b**) Molecular steps in the peptide self-assembly process: primary nucleation, nanofibril elongation, secondary nucleation via fragmentation, secondary nucleation via nanofibril surface nucleation [165]. Reprinted/adapted with permission from [165]. 2015, Arosio, P., et al.

The thermodynamics of the self-assembly process provides insights on the probability of the self-assembly reaction to occur. In contrast, the kinetics of self-assembly process determines how fast it proceeds [163]. The self-assembly process consists of primary nucleation, secondary nucleation, elongation, and fragmentation. Therefore, the kinetics of a self-assembly process leads to a sigmoidal curve and is characterized by lag-phase time ($\tau_{lag}$) and maximum growth rate ($v_{max}$), as sketched in Figure 4a [147,165,166,167,168,169,170,171,172,173].

The sigmoidal curve consists of three phases: the lag phase, the growth phase, and the equilibrium phase, as shown in Figure 4a [165,168]. The growth phase is also known as the elongation phase, and it is characterized as the transition zone with the highest slope. The growth phase is preceded and followed by the lag phase and equilibrium phase. The maximum speed of conversion of peptides to nanofibrils happens in the growth phase. The region before the growth phase is called the lag phase. The equilibrium phase presents the steady state stage of the fibrillation process, during which the peptide concentration reaches the equilibrium state. Previous works developed an empirical sigmoidal formula which successfully matched with the experimental findings.

Generation of nanofibrillar structures begins with the onset of nuclei formation (primary nucleation) and growth and generation of prefibrillar oligomeric species via thermal motion of peptides and their intermolecular forces [150,163]. At this step, the system is composed of various aggregated conformations including nanofibrillar structures, unordered aggregates, ring-like aggregates, and spherical aggregates [150]. Progress of peptide self-assembly consists of primary nucleation, nanofibril elongation, secondary surface nucleation, and nanofibril fragmentation, as sketched in Figure 4b. Nanofibril elongation is the mechanism in which the peptides attach to the ends of the preformed nanofibrils. Nanofibril fragmentation is the physical mechanism in which the free peptides detach from the nanofibrils or the nanofibrils are cut in between and are converted into more nanofibrils. Nanofibril fragmentation normally occurs due to agitation stress. Secondary surface nucleation is caused by association of the peptides on the surface of the nanofibrils.

During the self-assembly process, the primary nucleation and the nanofibril elongation result in a further fibril growth phase. Since growth of the nanofibrils normally occurs at the ends of the nanofibrils, nanofibril fragmentation has a critical role in the rate of the growth phase by increasing the number of available nanofibril ends for further elongation via attachment of the peptides to the ends. Secondary surface nucleation also is extremely crucial for the self-assembly process, especially at the quiescent condition, in which no agitation occurs in the system [165]. Self-assembly of the peptides continues until thermodynamic equilibrium is achieved [165,174].

(5) $$y_{Fibril}=y_{initial}+\frac{\alpha }{\left[1+exp\left(-v_{max}\left(t-t_{1/2}\right)\right)\right]}$$

In Equation (5), $y_{Fibril}$ denotes the temporal mass concentration of nanofibrils in the system, $y_{initial}$ represents the initial mass concentration of the nanofibrils in the system at the pre-transition base line. α is the amplitude of the conversion from the peptide condition to the nanofibrillar structure, $t_{1/2}$ represents the midpoint time during the growth phase, and $v_{max}$ denotes the apparent maximum growth rate. The lag phase time ($t_{lag}$) is determined by the following equation:

(6) $$t_{lag}=t_{1/2}-\frac{1}{2\;v_{max}}$$

In the sigmoidal curve of the nanofibril formation kinetics model, the lag phase time can be shown at the intersection of the line of the maximum growth rate with the time axis, as illustrated in Figure 4a.

Based on the Gibbs free energy curve of the peptide self-assembly process, the lag phase time depends on the time required for denaturing free peptides to aggregate and to create nuclei. According to the Gibbs free energy curve, nuclei exhibit the highest Gibbs free energy in the self-assembly process. Nevertheless, the nuclei are sufficiently stable to grow by more peptides in the self-assembly process towards the formation of nanofibrils.

The lag phase time shows how long the initial phase, which is the nuclei formation phase, takes. The lag phase time is inversely related to the rate of primary nucleation (faster primary nucleation has shorter lag phase time) [165]. In addition to primary nucleation, the lag phase time also depends on the rates of nanofibril elongation and the secondary nucleation process. Faster nanofibril elongation and secondary nucleation process lead to shorter lag phase. Vice versa, slower elongation and secondary nucleation cause a longer lag phase [165].

Maximum growth speed is the speed at which peptides self-assemble to create nanofibrils. Nanofibril elongation and the secondary nucleation process play a critical role in the maximum growth rate. Faster nanofibril elongation and secondary nucleation process lead to faster growth [165]. Previous works reported the roles of the rates of primary nucleation, secondary surface nucleation, and nanofibril elongation on the lag phase time and the speed of the growth phase [165]. As the speed of primary nucleation increases, the lag phase time is shorter, but there is no effect on the maximum speed of the growth phase. On the other hand, a higher rate of secondary surface nucleation leads to a faster growth phase, without any significant effect on the lag phase time. In contrast with these, a higher speed of nanofibril elongation leads to a shorter lag phase time as well as a faster growth phase.

The growth phase consists of the secondary nucleation process, nanofibril elongation, and nanofibril fragmentation. The onset of the primary nucleation process and the rate of fibrillation depend on several physical parameters: mechanical agitation strength, pH, ionic strength (size and type of salt), free peptide concentration, environment temperature, hydrophobic interface, and presence of preformed short nanofibrils in the peptide solution [151,168,175,176].

Several studies attempted to establish a general self-assembly kinetics model that includes the roles of all procedures of nanofibril formation: primary nucleation, secondary nucleation, nanofibril elongation, and nanofibril fragmentation [151,165,168,175,176,177,178,179,180]. The nanofibril formation process was found to be strongly dependent on the nucleation process [178]. This means that nuclei formation from free peptides takes longer than the elongation of the nuclei. Nanofibril fragmentation causes the formation of more available nuclei from the preformed nanofibrils [177,179,180,181]. 

The kinetics of the self-assembly process involves the influence of several fundamental physical molecular mechanisms including primary nucleation, secondary surface nucleation, nanofibril elongation, nanofibril dissociation, and nanofibril fragmentation. The speed of formation of nanofibrils with the desired fibril length depends on the rates of all of the aforementioned physical processes of the system as an infinite number of coupled non-linear partial differential equations [174,179,182,183,184,185].

It is critical to consider the fact that the physics of the nanofibril fragmentation is like the dissociation mechanism. The inverse of nanofibril fragmentation is known as association via connection of nanofibrils to each other. This process is very slow, as it requires the nanofibrils to structurally align in a specific way considering their respective orientations. Therefore, the inverse of nanofibril fragmentation is extremely unlikely to happen [174].

Nanofibril elongation (nanofibril growth phase) is characterized by a physical mechanism called the “dock-lock” process. In the “dock-lock” mechanism, peptides in a reversible fashion “dock” to pre-generated nanofibrils known as seeds in the system. This way, complex structures of peptide–seed conformations are formed [186,187]. The “docking” stage proceeds rapidly. After the “docking stage” finishes, the “docked” peptides go through a slow conformational change and they are irreversibly attached to the pre-generated nanofibrils (seeds) and consequently form the “locked” nanofibril conformations. Thereafter, the “locked” nanofibril conformations proceed further to serve as seeds for attachment of more peptides. The “docking-locking” process continues until all peptides incorporate in this physical process and the system reaches equilibrium.

The speed at which the peptides attach to the nanofibrils is denoted by $k_{+}$ or $k_{on}$, which is called association rate. The speed at which the peptides detach from the nanofibrils is shown by $k_{-}$ or $k_{off}$ and is called dissociation rate. An equilibrium state is reached after giving sufficient time for peptides to attach to the nanofibrils in the system. The concentration of peptides at equilibrium (${\left[P\right]}_{\infty }$) which are found to be free and not associated with any nanofibril is applied to quantify the ratio of the speed of dissociation over the speed of association, which is known as the equilibrium dissociation constant and denoted by $K_{D}$ [188], as defined by Equation (7):

(7) $$K_{D}=\frac{k_{-}}{k_{+}}=\frac{k_{off}}{k_{on}}=\frac{{\left[P\right]}_{\infty }}{\psi }$$

in which ψ presents the standard reference peptide concentration (ψ=1 M). Accordingly, the equilibrium association constant, $K_{A}$, is defined as the inverse of the equilibrium dissociation constant, as shown in Equation (8):

(8) $$K_{A}=\frac{1}{K_{D}}=\frac{k_{on}}{k_{off}}=\frac{\psi }{{\left[P\right]}_{\infty }}$$

Thermodynamics and kinetics of peptide self-assembly strongly depend on several intermolecular physical interactions: hydrogen bonding networks between peptides, noncovalent pi–pi stacking of aromatic rings of hydrophobic (nonpolar) residues of the peptides, electrostatic interactions between charged/polar residues of the peptides and between charged/polar residues of the peptides and aqueous environment, hydrophobic interactions between hydrophobic residues of the peptides, and van der Waals intermolecular interactions between molecules of the peptides [189]. pH, ionic strength and temperature of the aqueous environment, as well as the types of the residues of the peptides (i.e., hydrophobic, negatively and positively charged, and polar) could significantly influence the kinetics and thermodynamics of the peptide self-assembly process.

As Gibbs free energy of native free peptides is higher than Gibbs free energy of their ordered aggregate forms (nanofibrils), peptides exhibit a higher tendency to self-assemble. Therefore, the higher Gibbs free energy difference between native peptides and nanofibrillar structures promotes more and easier peptide self-assembly, which leads to a faster peptide self-assembly process.

## 4. Pathophysiology of Self-Assembled Peptides

### 4.1. Biophysics of Self-Assembled Peptides in Physiology

Elasticity of the protein backbone structure and its side chain orientation allows proteins to form a stable highly compact three-dimensional conformation by reaching the minimum free energy level. Stable interactions between hydrophobic residues of protein amino acid sequences to cover hydrophobic residues from an aqueous environment are the main physical force to reach the total minimum free energy level. Proteins naturally like to fold into a highly complex and compact three-dimensional stable conformation. A biologically active protein consists of one or more peptides. A peptide has several amino acids connected to each other by peptide bonds. A peptide bond is a chemical bond between an amine side of an amino acid and a carboxyl side of another amino acid along the peptide backbone.

In healthy biological conditions, proteins strongly tend to fold into a native and desired configuration. The native conformation is the most stable configuration in the physiological state considering the cellular environment in terms of existence of molecular chaperones and proteolytic enzymes [190]. Under certain conditions, an alternative minimum free energy level (*G*) landscape can be reached via molecular interactions including hydrophobic/hydrophilic forces and hydrogen bonds between amino acid side chains, which induce the proteins to go through a physio-chemical procedure known as self-assembly.

### 4.2. Protein Misfolding

Protein self-assembly could fail to maintain proteins being properly folded into a desired conformation. This leads to a protein misfolding and generation of highly ordered aggregates known as nanofibrils [141,191,192]. Protein misfolding induces failure of a protein to adopt and maintain its native desired biological state, and it consequently forms toxic compounds which are extremely harmful for physiological function [141].

### 4.3. Self-Assembled Peptides in Disease 

Several chronic disorders relate to protein misfolding including neurodegenerative complications such as Alzheimer’s, Parkinson’s, dementia with Lewy bodies, Huntington’s. Other examples of chronic complications are nonneuropathic systemic amyloidosis including AL amyloidosis and lysozyme amyloidosis and nonneuropathic localized disorders such as T2DM and pulmonary alveolar proteinosis [141]. However, a vast number of studies reported that the toxicity of the protein misfolding process is not connected to the bulk region of a dysfunctional aggregate but to the transient generation of toxic oligomers [163]. Toxic oligomers are induced by undesired physiologic exposure of amino acids of the protein structure that would be protected inside the folded configuration [141].

## 5. Applications of Molecular Self-Assembly in Bio-Nanotechnology

Self-assembled peptides in nanofibrillar structures have remarkable stability against undesired physical, chemical, and biological conditions. These outstanding features of the nanofibrils make them the best candidates in nanotechnology [105,193]. Nanofibrils were initially recognized as the principal reason for various chronic neurological complications including Alzheimer’s, Parkinson’s, dementia with Lewy bodies, and Huntington’s [141]. Therefore, nanofibrils were not recognized as promising drug formulations and drug delivery systems [141].

Multiple studies revealed that precursors of nanofibrils known as oligomers are the main pathogenic species that could lead to neurological disorders [105,141]. Recent studies explored various functional biological applications of nanofibrils including bacterial coatings and scaffolds, which shed light on the promising application of peptide self-assembly in tissue regeneration [110]. For instance, there were multiple attempts that proved the reversible generation of nanofibrils from cellular proteins including tubulin and actin in the human body [111].

**Figure 5:**
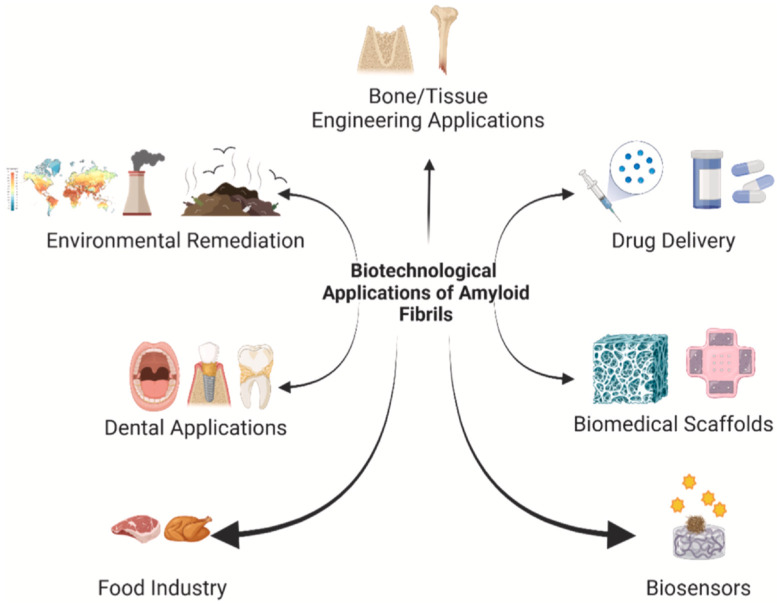
Applications of the biological self-assembled nanofibrils for biomedical purposes [197]. Highly ordered nanostructures (nanofibrils) with strong regulatory monitoring show tremendous advantages in various fields of biomedicine: tissue engineering, biological scaffolds, drug delivery systems, dentistry, food safety, biosensors. Reprinted/adapted with permission from [197]. 2024, Afjadi, M.N., et al.

Several works illustrated that human peptides can self-assemble into nanofibrils for advancement in nanomedicine. Therefore, these natural nanofibrils could be recognized for various biomedical and nanotherapeutic purposes including smart drug delivery systems in the form of micro-capsules and nano-capsules and engineered tissue matrices such as hydrogels. Figure 5 illustrates some of the applications of nanostructured self-assembled peptides into nanofibrils in various bio-nanotechnologies. The nanofibrils, generated from human peptides, were shown to be the best promising candidates for long-acting drug formulations. This is because the nanofibrils exhibit long bioactive characteristics based on their extraordinary biocompatibility, bio-chemo-physical stability, and regulated site-specific drug delivery effects [112,113,114,115,116].

Despite adverse effects of protein misfolding which generate nanoscale amyloid fibrils, a vast number of highly ordered nanofibrils showed constructive physiological influences [105]. For example, the bacteria *Escherichia coli* consists of extracellular nanofibrils, known as curli, for cell–cell interactions to maintain host colonization [105,194]. Another example is Human protein Pmel17, which forms a nanofibrillar structure, extremely important for skin pigmentation progress [105,195]. Another instance is the nanofibrillar structure from protein chorion in eggshell, which protects the oocyte and keeps the embryo away from any undesired and unpredictable environmental harm [196].

**Figure 6:**
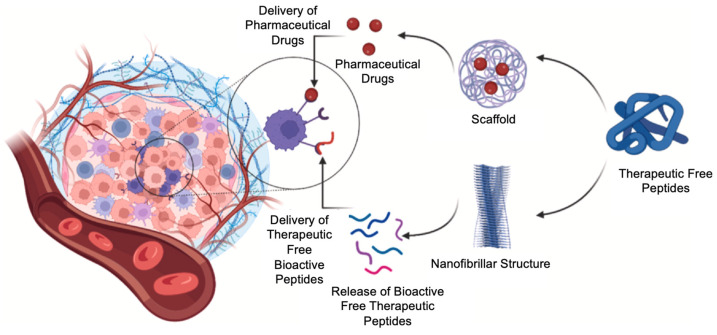
Application of self-assembled peptides into highly organized nanofibrils as a smart drug delivery system. Nanofibrils can be generated via self-assembly of peptides under certain physical conditions [197]. Upon administration, the nanofibrils can release bioactive peptides from the ends of the nanofibrils. This enables the highly controlled and sustained release of pharmaceutic peptides into the body to induce their pharmacological effects at the target site. Reprinted/adapted with permission from [197]. 2024, Afjadi, M.N., et al.

These studies showed the constructive and desired role of nanofibrils as potential stable drug depots for prolonged pharmaceutical drug formulations with highly controlled release mechanisms of biologically active peptides in vivo [105,106]. Peptides, small proteins, can self-assemble into highly ordered nanofibrillar structures without being folded. For example, peptides form bioactive highly ordered nanofibrillar structures to store pituitary peptide hormones in secretary granules [106]. Generating highly ordered nanofibrillar structures strongly depends on the amino acid sequence and selected peptide hormone, which induces the fact that the granule core of the nanofibrillar structure only consists of selective peptide hormones [106]. Figure 6 illustrates the bio-physical mechanism of the function of the two self-assembled smart nanostructures known as the nanofibrils and the amyloid scaffolds to deliver the pharmaceutical agents (drugs) in vivo at the target site at a sustained and regulated manner.

## 6. Type 2 Diabetes Mellitus (T2DM): Pathology and Treatment Methods and Their Challenges

### 6.1. Pathology of Type 2 Diabetes Mellitus (T2DM)

Diabetes is considered as the most prevalent chronic disorder, with 462 million people in 2017 as the top 10 causes of death in the world [117,118,119]. Due to extremely fast population growth, sedentary lifestyle, and poor diet, this estimate is predicted to increase to 592 million by 2035 [117]. Diabetes has two types: type 1 diabetes mellitus (T1DM) and type 2 diabetes mellitus (T2DM) [120]. T1DM is generated by no or negligible insulin secretion from the pancreas, and it counts for 10% of diabetic patients [120]. T2DM is caused by insufficient insulin secretion from the pancreas, and it counts for 90% of diabetic patients [120]. There are several factors for the epidemic of T2DM: genetics and hereditary, age, obesity, sedentary lifestyle, poor diet, smoking, and hypertension [119].

In healthy individuals, plasma glucose level is normally regulated by secretion of glucagon from pancreatic *α* cells and secretion of insulin from pancreatic β cells. As glucagon derives gluconeogenesis and glycogenolysis in the liver and muscle cells, insulin prevents glucagon’s influence by up-taking plasma glucose to insulin-sensitive tissue cells [120]. This biological process is controlled by a feedback loop system between insulin-sensitive tissue cells and pancreatic β cells [121]. Pancreatic β cells secrete insulin to mediate uptake of plasma glucose and fatty acids to insulin-sensitive tissue cells, and in response to this biological process, insulin-sensitive tissue cells send signals to pancreatic β cells for more insulin secretion [121].

Prediabetes and T2DM are generated by reduced insulin sensitivity (also known as insulin resistance) in tissue peripheral cells. As insulin resistance of the peripheral cells results in rising insulin secretion from pancreatic β cells, the insulin insensitivity of the tissue peripheral cells does not prompt any alarming symptom. However, incapability of pancreatic β cells to persistently secrete enough insulin promotes high glucose level in the blood [121]. Prediabetes is due to impaired fasting glucose (IFG) and impaired glucose tolerance (IGT) [122]. Continued IFG, IGT, and hyperglycemia promote reduction of the pancreatic β cells [123]. Furthermore, uncontrolled secretion of glucagon from pancreatic α cells, especially after food intake, causes rise of glucagon level and enhanced hyperglycemia [121].

Pathology of T2DM, due to permanent elevated blood glucose level, is vascular disease via undesired changes in the structures of the blood vessels, microaneurysms, neuropathy, and chronic kidney disease. Hyperglycemia due to IFG and IGT can promote risk of severe clinical cardiovascular disease [122]. Due to pathological impacts of T2DM, life-time treatment of T2DM patients is extremely necessary [119].

To address this significant life-threatening challenge in the daily life of T2DM patients, various therapeutic approaches were investigated. These therapeutic methods were also applied on T2DM patients to treat this chronic disorder. The next section (Section 6.2) will provide a summary of the highlighted advancements in treatment of T2DM.

### 6.2. T2DM: Treatment Methods

Weight reduction is considered to be a fruitful way to treat T2DM. However, intensified plasma glucose excretion in urine causes lower urinary tract infection and cardiovascular complications. Therefore, severe hyperglycemia extremely requires pharmaceutical intervention to increase insulin levels in the blood. 

Daily oral administration of sulfonylurea antidiabetics derives a series of biological signaling in pancreatic β cells to enhance insulin secretion. If this does completely resolve hyperglycemia to regulate the glucose level, additional human insulin is injected subcutaneously to the T2DM patients [124]. Daily subcutaneous administration of human insulin causes patient discomfort, and it is highly expensive.

Moreover, consistent plasma glucose reduction by daily insulin injection and daily oral administration of sulfonylurea antidiabetics causes weight gain, severe hypoglycemia, and possible cardiovascular complications [124,125]. The most effective treatment method for T2DM and obesity patients is bariatric surgery, known as the Roux-en-Y Gastric Bypass (RYGB) surgery. RYGB induces remarkable changes in the expression levels of certain gut peptide hormones. This biological finding recommends the further advancement of incretin-based nanomedicine techniques for treating obesity and T2DM patients [126].

After energy intake, the gut secreted four peptide hormones during the postprandial stage for food ingestion. These peptides are called gastric inhibitory peptide (GIP), peptide tyrosine–tyrosine (PYY), glucagon-like peptide 1 (GLP-1), and oxyntomodulin (Oxm). These peptide hormones induce satiety feeling, prevent gastric exocrine secretion, slow down gastric emptying, and promote nutrient uptake to the peripheral cells.

Appetite is impeded by sending signals to the central nervous system via endocrine and pneumogastric nerves [127]. Hindering gastric emptying induces appetite suppression and consequently prevents further food intake. These peptides also play a significant role in the function of pancreatic cells in secretion of glucagon and insulin.

GIP is a 42 amino-acid peptide and is secreted during the postprandial stage from intestinal K cells [126]. Its metabolic activities are enhancement of insulin secretion, reducing glucose tolerance in T2DM patients, and satiety feeling [126]. Its metabolic activities are degraded by the proteolytic activation of dipeptidyl peptidase-4 (DPP-4). GIP increases insulin secretion from pancreatic β cells but also reduces glucose tolerance in T2DM patients.

The PYY peptide hormone consists of 36 amino acids, with tyrosine residues at the C-terminus and N-terminus from the pancreatic polypeptide family [128]. PYY is released during the postprandial stage from intestinal L cells. Its secretion amount strongly depends on food intake. PYY does not stay active longer than 6 h [128]. PYY induces appetite suppression. PYY does not have any role in insulin and glucagon releases [128]. PYY metabolic function can be degraded by DPP-4.

GLP-1 has 29 amino acids. GLP-1 is a C-terminally amidated peptide hormone [126]. GLP-1 is secreted during the postprandial process from neuroendocrine L cells of the gut after energy intake. The GLP-1 secretion is through prohormone proglucagon cleavage from gene GCG. GLP-1 expression and secretion level are proportional to food intake [126,129]. GLP-1 enhances insulin release, reduces glucagon secretion, improves glucose tolerance in T2DM patients, hinders gastric emptying, suppresses appetite, and rises energy spending [126]. GLP-1 becomes inactivated by the proteolytic degradation role of DPP-4 and Neprilysin.

Oxm is a gut peptide hormone with 37 amino acids. Oxm is expressed from a proglucagon precursor and is a produce of glucagon gene GCG. The GCG gene is expressed in the intestine, pancreas, and central nervous system. Oxm consists of glucagon’s 29 amino acid sequence followed by 8 amino acids with carboxy-terminal extension [128,129]. Oxm is secreted during the postprandial stage from intestinal L cells. The Oxm secretion amount is proportional to the amount of food ingestion [126,128]. Oxm increases insulin release, reduces glucagon release, improves glucose tolerance in T2DM patients, suppresses appetite, and enhances energy expenditure [126]. Oxm’s longest in vivo bioactivity is approximately 30 min after food intake, and its metabolic functions become stopped by the proteolytic degradation effect of DPP-4 and Neprilysin [128].

GLP-1, Oxm, and glucagon genes are transcribed in the proglucagon gene GCG. In this transcription process, the proglucagon gene goes through an enzymatic stage in which it generates peptide hormones based on each specific tissue. This means that the proglucagon gene expresses different peptide hormones from the same protein based on the related tissue [129]. In pancreatic *α* cells, prohormone convertase 2 (PC2) produces glucagon as a major peptide hormone. Prohormone convertase 1 and 3 (PC 1/3) mainly generate glicentin, Oxm, GLP-1, and GLP-2 as major peptide hormones of gene GCG in intestinal L cells of the gut and central nervous system [129].

The main metabolic function of these peptide hormones is during the postprandial stage by appetite suppression, delaying gastric exocrine release, and expediting nutrient ingestion to the peripheral cells [127]. After RYGB, T2DM and obese patients showed enhanced release of PYY, glucagon, GLP-1, and Oxm from intestinal cells [126]. This fascinating finding highlighted the fact that pharmaceutical drug formulations based on these peptide hormones present a promising therapeutic effect on T2DM and obesity.

Although, these four gut hormones promote satiety feeling, their role on plasma glucose is very different [127,128]. PYY does not have any effect on glucose and insulin and glucagon release. GIP increases insulin release in pancreatic β cells, but it reduces glucose tolerance in T2DM patients. GLP-1 and Oxm enhance insulin secretion and reduce glucagon release [120].

GLP-1 administers its metabolic function via a single receptor known as GLP-1R. GLP-1R is expressed throughout the entire human body. Activation of GLP-1R has effects on pancreatic *α* cells and pancreatic β cells to regulate normal plasma glucose level during the postprandial stage. In general, GLP-1R agonists reduce glucose level in T2DM patients where treatment method by sulfonylurea antidiabetics administration is not effective [130]. GLP-1R activation in the hypothalamus induces sensation of satiety [127].

Many GLP-1 analogues induce weight loss in healthy individuals and patients with obesity and T2DM [131,132]. Weight loss achieved by feeling of satiety is hindered by damped energy expenditure through activation of GLP-1R in cardiac tissues [126]. They also reported that GLP-1 analogues cause discomforting side effects such as nausea and vomiting for patients [120,126].

Oxm consists of the complete amino acid sequence of glucagon and an additional 8 amino acids with carboxy terminal extension [129]. Oxm activates two receptors: GLP-1R and glucagon receptor (GCGR). However, Oxm has a lower effect on activation of both receptors in contrast with activation of each receptor with their corresponding single-receptor agonist. For instance, GCGR activation by glucagon or GLP-1R activation by GLP-1 are much more effective that their activations by Oxm [127,133].

Oxm’s dual receptor agonism induces the antidiabetic role of GLP-1 and stronger weight loss effect of GLP-1 [130,134]. This is attributed to the additional satiety sensation caused by glucagon effort while increasing energy spending by GCGR activation [135,136]. As a result, the concomitant role of glucagon and GLP-1 was shown to be more effective in treatment of T2DM and obesity in contrast with single GLP-1 or glucagon agonism [130].

In vivo and preclinical studies reported strong appetite suppression and energy intake reduction after subcutaneous (s.c.) injection of Oxm [137,138]. Clinical studies showed substantial weight loss in patients with obesity or T2DM by consistent Oxm s.c. administration [128,139]. Moreover, there was no sign of discomforting side effects such as nausea or vomiting by administration of Oxm in patients.

Exenatide, Liraglutide, Lixisenatide, and Dulaglutide, analogues of GLP-1, were also approved as prolonged bioactive drug formulations for treatment of patients with T2DM [126]. Nonetheless, GLP-1, its analogues, and PYY induce discomforting side effects such as nausea and vomiting in patients [120,126]. By comparison, Oxm and its analogues did not have any side effects. The Oxm analogue, generated by non-proteogenic amino acid substitution in its position 2 of amino acid sequence, was reported to be an extremely prolonged bioactive drug for weight loss and glucose control in T2DM patients [134,138].

### 6.3. T2DM: Challenges in Current Treatment Methods

Gut peptides are only active for a short time during the postprandial stage. After that, their plasma half-lives and metabolic functions become proteolytically stopped. GLP-1 and Oxm are degraded by the proteolytic role of DPP-4 and Neprilysin [137,140]. Multiple strategies were studied to improve plasma half-life of the circulating peptide hormones in the blood. One technique was prevention of proteolytic effect of enzymes by peptide analogues that showed resistance to the enzyme’s proteolytic degradation role. The other technique was forming a nanofibrillar conformation of the peptide hormone as a prolonged smart reversible drug delivery system in the body. Impeding the proteolytic effect of DPP-4 can enhance plasma half-life of GLP-1 and Oxm. This increases the level of insulin production, and glucagon secretion is hindered, effectively leading to glycemic control in patients with T2DM.

Inhibition of DPP-4 improves inherent levels of GLP-1 and Oxm. These DPP-4 inhibitors enhanced insulin production and prevented glucagon secretion in the body. This way, the DPP-4 inhibitors normalize glucose levels. The DPP-4 inhibitors are Sitagliptin and Vildagliptin. They were approved as pharmaceutical drug formulations to treat T2DM by recombinant administration of other antidiabetic therapies. Despite their promising role in glucose control, the influence of these DPP-4 inhibitors on other peptides, such as the peptide hormone PYY, hinders its effective role on weight loss, which is extremely critical for patients with obesity and T2DM [120,125].

Prolonged bioactive GLP-1R agonists were the subject of intense pharmaceutical studies over the last several decades. These studies concluded with several extraordinary successful antidiabetic drug candidates and one incretin peptide hormone analogue approved for weight loss. Therefore, there are nine approved GLP-1R agonist therapeutics. Two of these drug formulations also contain insulin.

Approaches to hinder the DPP-4 proteolytic degradation role include steric slow down by acylation (Liraglutide) and substitution of Ala7 for Glycin such as Exenatide, Lixisenatide, Albiglutide, and Dulaglutide. Another strategy was the nonproteinogenic amino acid Amino-isobutyric acid (Aib) substitution (Semaglutide). Acylation-driven albumin binding (Liraglutide, Semaglutide), albumin fusion (Albiglutide), or IgG4 Fc fusion (Dulaglutide) could strongly increase plasma half-life by lowering the glomerular filtration and hepatic degradation.

Dosing frequency ranges from daily twice to weekly once, with tendentially better glucose regulation reached by prolonged bioactive drug formulations [130]. Moderate weight loss by GLP-1R agonists was reported in healthy individuals and obese patients and T2DM patients [106,196]. The effect of weight reduction attained by appetite suppression is strongly required by lowering the energy spending induced by GLP-1R activation in cardiac tissue [129]. Several studies reported that hindered gastric emptying process does not have a prolonged beneficial role due to habituation [140]. There were several reports of nausea, vomiting, and diarrhea as frequent adverse side effects of all GLP-1R agonists [125,129,130].

## 7. T2DM: Treatment by Self-Assembled Peptide Hormones

The most recent study reported the promising application of the molecular self-assembly of oxyntomodulin peptides to generate smart highly ordered nanostructures (nanofibrils), as a self-mediated drug delivery route, for a prolonged, safe, and highly effective treatment of T2DM [99,198,199,200]. They showed that the subcutaneous injection of the oxyntomodulin nanofibrils into the T2DM subjects (mice) exhibits a prolonged, safe, and smart drug delivery route for the pharmaceutically bioactive oxyntomodulin peptides in vivo [99,198,199,200].

They also investigated the in vivo pharmacokinetic and pharmacodynamic profiles of the oxyntomodulin self-assembled nano-scale fibrils on animal subjects (rats) [99,198,199]. They reported a prolonged exposure of the oxyntomodulin in serum for at least 5 consecutive days in contrast with subcutaneous injection of free oxyntomodulin peptides, which were only bioactive in serum up to approximately 4 h [198]. This recent outstanding finding offers a great promise of application of the gut peptide hormone self-assembly into the smart nano-thickness fibrillar structures for prolonged and safe treatment of T2DM.

There were significant efforts to apply the lipidation technique to generate nanofibrillar structures from numerous pancreatic polypeptide families including peptide YY (PYY). The pancreatic polypeptide families are released from the gastrointestinal tract after food ingestion. These gut peptide hormones can have vital roles in metabolism, anti-obesity, and treatment of type 2 diabetes mellitus [201,202,203,204,205,206]. PYY is released from the L cells of the gastrointestinal tract after food intake and exhibits high affinity for the Y_2_ receptor. The Y_2_ receptor is related to the control of food ingestion and gastric emptying [206]. Therefore, PYY self-assembly could prolong the therapeutic effects of PYY and enhance patient compliance for individuals with T2DM.

The peptide hormone self-assembly has numerous advantages including improved adsorption, distribution, metabolism, and excretion (ADME) profiles. The self-assembled peptides in nanofibrillar structures have outstanding regulated sustained-release capability, which results in a controlled prolonged drug exposure duration, enhanced bioavailability, and persistent bioactivity of the drug for patients. In contrast, the conventional administration of free native peptides fails to do so. The subcutaneous administration of nanofibrils (free bioactive native peptides depot) can facilitate this outstanding advantage of sustained prolonged drug delivery for T2DM patients.

Oxyntomodulin nanofibrils showed extraordinary controlled slow sustained release of free bioactive peptides from subcutaneous space into the serum. The oxyntomodulin nanofibrils showed significant stability of released bioactive oxyntomodulin peptides in the serum for days as compared to the administration of free peptides in a conventional manner [109]. A previous study reported the excellent therapeutic efficacy of the oxyntomodulin nanofibrils for treatment of T2DM [109,198]. One of the main reasons to apply gut peptide hormones to generate nanofibrils for treatment of T2DM is their safety characteristics, which significantly reduce the off-target side effects. 

The pharmacological effect of oxyntomodulin nanofibrils, in vivo, via monitoring the blood glucose level and body weight in mice after subcutaneous administration of free bioactive oxyntomodulin and oxyntomodulin nanofibrils, showed the enhanced prolonged bioactive presence of oxyntomodulin in the serum for lowering the blood glucose level and body weight in mice [198]. The subcutaneous administration of oxyntomodulin nanofibrils in rats leads to excellent prolonged exposure in the serum with sustained oxyntomodulin bioactivity presence in the serum for at least 5 days as compared to the conventional administration of free oxyntomodulin peptides, which were not detectable in the serum after a few hours [198]. That result showed the promising application of oxyntomodulin nanofibrils as a prolonged pharmacological controlled bioactive peptide-release depot for long-term controlled treatment of T2DM [198]. The bioactivity and cytotoxicity of the released oxyntomodulin peptides from the nanofibrils were also studied in vitro [198]. They reported that the free oxyntomodulin peptides, released from the oxyntomodulin nanofibrils, exhibited an outstanding full potency to GLP-1 and glucagon receptors, which promote blood glucose reduction and increased energy expenditure in the body [198]. Moreover, the in vitro cytotoxicity of the free bioactive oxyntomodulin peptides, released from the oxyntomodulin nanofibrils, was assessed by measuring the metabolic bioactivity of the living cells. They found that free bioactive released oxyntomodulin peptides were not cytotoxic [198]. A similar cytotoxicity test was conducted on oxyntomodulin nanofibrils, and they found non-cytotoxic characteristics of the nanofibrils [198].

There have been preclinical studies (animal studies) to assess the safety and efficacy of oxyntomodulin nanofibrils in mice to obtain pharmacokinetic and pharmacodynamic profiles of oxyntomodulin nanofibrils [198]. However, no clinical studies have yet been conducted on human subjects to assess the safety and efficacy of oxyntomodulin nanofibrils for pharmacokinetic and pharmacodynamic assessment. The clinical study on oxyntomodulin nanofibrils must be conducted soon. The clinical potential of oxyntomodulin nanofibrils will be only fully realized once their physicochemical, pharmacokinetic, and pharmacodynamic properties are precisely regulated on human subjects.

Despite the promising influence of oxyntomodulin on body weight reduction, energy expenditure rise, and blood glucose lowering to treat T2DM, its metabolic effects become degraded through proteolytic degradation in the serum by proteases such as DPP-4, which limits the pharmacological activity of the free oxyntomodulin peptides upon release from the nanofibrils [109]. To address this challenge, a recent study has applied molecular self-assembly on an oxyntomodulin analogue. A recent study found that a discrete change to the amino-acid sequence of oxyntomodulin will promote its resistance against DPP-4 enzymatic degradation and will increase the serum stability of the free peptides upon release from the nanofibrils. In the recent study, the serin residue in position 2 of the oxyntomodulin amino acid sequence was replaced by the aminoisobutyric acid (Aib) residue. The Aib2-oxyntomodulin was shown to be resistant to DPP-4 proteolytic degradation [109]. In contrast with oxyntomodulin nanofibrils, Aib2-oxyntomodulin nanofibrils were shown to have higher presence in the serum due to faster release of the free bioactive Aib2-oxyntomodulin peptides from the subcutaneous administered nanofibrils in mice [109]. The efficacy, safety (cytotoxicity profile), and pharmacokinetic profiles of Aib2-oxyntomodulin nanofibrils were investigated through in vitro cell line assays and in vivo animal studies in mice [109]. They found that Aib2-oxyntomodulin nanofibrils exhibit great potency to the human GLP-1 receptor (GLP-1R) and human glucagon receptor (GCGR). This finding revealed the metabolic effects of Aib2-oxyntomodulin nanofibrils to reduce food intake and increase energy expenditure in mice [109]. However, the molecular self-assembly process of Aib2-oxyntomodulin peptides into nanofibrillar structures displayed a slower speed compared to the rate of the oxyntomodulin self-assembly process [109,198].

## 8. Conclusions and Discussion

The application of human peptides as a medicinal drug is going through a revolution in the field of pharmacology. It is expected to achieve revolutionary drug formulations to treat T2DM with extraordinary, enhanced safety and efficacy profiles, which could ultimately improve patient compliance. The recent experimental research just offered a proof of concept of the reversible spontaneous molecular self-assembly process to form structurally organized nano-thickness fibrils as smart nanostructures to deliver the human gut peptide hormone, oxyntomodulin, in vivo for prolonged and safe treatment of T2DM and obesity [99,198,199,200].

The nanofibril was considered as a good strategy to enhance the short plasma half-life of the gut peptide hormone in vivo. This state-of-the-art approach could reduce the frequency of subcutaneous injection of the pharmacological gut peptide hormone in vivo for T2DM patients, and, therefore, it could significantly reduce cost and patient discomfort.

Despite all these beneficial factors of the peptide self-assembly process to generate smart nanostructured drug delivery systems for treatment of T2DM, many questions are still unanswered. Until these concerns are resolved, this state-of-the-art pharmaceutical approach cannot be applied in human subjects. Following is the list of concerns related to the applicability of this pharmacological technique:

1. The molecular structures of these nanofibrils are still not fully resolved. It is extremely vital to have completely uniform nanofibrils in terms of length, structure, and thickness. And so forth, the nanofibrils can release the bioactive peptides in a controlled and precise manner in vivo. This concern intends to avoid adverse pharmacological effects of this revolutionary drug delivery route such as hypoglycemia and hyperglycemia in T2DM patients.
2. It is still not clear if the generated nanofibrils could overcome severe physical conditions including harsh temperature variation, abrupt mechanical environment such as agitation and container’s surface hydrophobicity, etc. The nanofibrils might undergo alternative conformations due to these environmental condition changes, which can consequently influence the efficacy and safety profiles of the drug delivery route when administered into patients in vivo.
3. This technique has not yet been applied on human subjects. After the first challenge is fully resolved, the safety and efficacy of this smart drug delivery method must be tested on human subjects. The clinical possibility of the smart nanofibrils can only be completely recognized once the physicochemical, biochemical, pharmacokinetic, and pharmacodynamic features of the nanofibrils are precisely regulated in vivo on human subjects.

## 9. Future Research Directions

Various gut peptide hormones need to be explored to study their tendency to self-assemble into nanofibrils. This broad investigation might shed light on the discovery of better human-derived gut peptide hormone candidates in terms of efficacy and prolonged activation via their self-assembly into nanofibrillar structures.

Advanced biosensing experiments need to be conducted to gain deep insights on the role of various physical parameters on the kinetics and thermodynamics of the self-assembly process of various native human gut peptide hormones. These studies will improve the knowledge to generate the stable and precisely regulated nanofibrils as a smart nanostructured drug delivery system for a prolonged treatment of T2DM. The physical parameters that need to be extensively studied are pH, size of salts, type of salts, environment temperature, mechanical agitation, and pressure. These experimental studies must be conducted with highly precise and advanced biosensors such as quartz crystal microbalance with dissipation (QCMD), thioflavin T (ThT) fluorescence spectroscopy, and isothermal titration calorimetry.

A comprehensive study on the kinetics of peptide release from nanofibrils must be considered under various physiological conditions. This investigation can be accomplished by a very precise biosensing technique known as dual polarization interferometer (DPI) and highly resolved imaging methods including atomic force microscopy (AFM) and cryogenic electron microscopy (cryo-EM) for observation of findings from DPI. 

DPI is a surface-based biosensing technique to evaluate the dissociation profile of the nanofibrils under various biochemical conditions mimicking physiological pH, temperature, and ionic strength, while nanofibril are in direct interaction with subcutaneous space condition. DPI measures the real-time dissociation profile of the nanofibrils by monitoring the changes in thickness and refractive index of the layers of the nanofibrils deposited on top of the collagen-coated biosensing surface. Therefore, the DPI technique could provide significant insights on the profile of bioactive peptide release from the nanofibrils in physiological condition. AFM and cryo-EM provide precise information regarding atomic-level and near-atomic-level resolution of the structures of the nanofibrils. The dissociation profile of the nanofibrils under various storage and transport conditions including room, fridge, and freezer can be examined by the NanoDrop spectrophotometer, and the results can be verified by imaging atomic and near-atomic-level structures of the nanofibril using AFM and cryo-EM.

It is extremely vital to determine the types of the external stimuli that affect the peptide release characteristics and the level of their influence. The externals that might regulate the release profile of the peptides from the nanofibrils are listed as environment temperature, pH, types of salts, sizes of the salts, excipients, presence of in vivo binding components, etc. In vivo pharmacokinetic and pharmacodynamic studies must be conducted to validate the stability and long bioactivity of various gut peptide hormone-derived nanofibrils in vivo.

It is very important to find an optimum condition in which the nanofibrils do not show polymorphism. The polymorphic feature of nanofibrils could have an adverse pharmacological effect of the peptides on the target site of the T2DM patients. These adverse side effects due to polymorphism could be hypoglycemia or hyperglycemia, which both are highly undesired for T2DM patients.

Although no significant adverse effects were reported through previous in vivo studies, it is extremely vital to further explore the potential safety concerns of self-assembled peptide nanofibrils for safe drug delivery approach. Give that nanofibrils may behave in a different manner than native free bioactive peptides, it is very important to expand the studies associated with the long-term toxicity and immunogenicity of these novel drug formulations. The examples, that should be extremely considered, are the biodegradation of packaged nanofibrillar structures and possible accumulation in tissues. Moreover, the ability of maintaining the quality and stability of self-assembled peptide nanofibrils for long duration of production and storage at various environmental conditions must be examined.

Further studies need to be conducted on how to optimize the process of formation of nanofibrils in terms of shelf-life, stability in harsh environmental conditions, abrupt changes in storage conditions, and various storage conditions. Moreover, there must be a comprehensive study on generating a roadmap for robust manufacturing condition(s) to optimize the speed of the peptide self-assembly process to form stable nanofibrils with long shelf-life via considering various physical factors including pH, temperature, pressure, and container surface chemistry/geometry/structure. Furthermore, extensive studies need to be conducted on optimizing the drug release and absorption profiles in vitro and in vivo. There have not been any clinical studies on the assessment of physicochemical, pharmacokinetic, and pharmacodynamic profiles of nanofibrils including oxyntomodulin nanofibrils for treatment of T2DM. The clinical potential of application of gut hormone peptide self-assembled nanofibrils in treatment of T2DM will be only completely recognized when the safety and efficacy of such nanofibrils are precisely regulated in human subjects.

To achieve the ultimate goal, which is to consider the regulatory and commercialization aspects of gut peptide hormone self-assembled nanofibrils as smart controlled prolonged drug depot (nanofibrils) for treatment of T2DM, academia and industry need to collaborate in a very efficient and parallel manner to explore regulatory approval pathways and overcome potential barriers to patient adoption, among other objective issues related to patient compliance.

## Data Availability

There are no new data in this review article.
